# Queen Nefertari, the Royal Spouse of Pharaoh Ramses II: A Multidisciplinary Investigation of the Mummified Remains Found in Her Tomb (QV66)

**DOI:** 10.1371/journal.pone.0166571

**Published:** 2016-11-30

**Authors:** Michael E. Habicht, Raffaella Bianucci, Stephen A. Buckley, Joann Fletcher, Abigail S. Bouwman, Lena M. Öhrström, Roger Seiler, Francesco M. Galassi, Irka Hajdas, Eleni Vassilika, Thomas Böni, Maciej Henneberg, Frank J. Rühli

**Affiliations:** 1 Institute of Evolutionary Medicine, University of Zurich, Zurich, Switzerland; 2 University of Turin, Department of Public Health and Paediatric Sciences, Legal Medicine Section, Turin, Italy; 3 UMR 7258, Laboratoire d’Anthropologie bio-culturelle, Droit, Etique & Santé (Adés), Faculté de Médecine de Marseille, Marseille, France; 4 University of York, Department of Archaeology, York, United Kingdom; 5 BioArCh, Departments of Archaeology, Biology & Chemistry, University of York, York, United Kingdom; 6 University Hospital Zurich, Department of Radiology, Zurich, Switzerland; 7 Ion Beam Physics. Labor f. Ionenstrahlphysik (LIP), ETH Zürich, Zurich, Switzerland; 8 Fondazione Museo Egizio of Turin, Turin, Italy; 9 Medical School, University of Adelaide, Adelaide, Australia; Seoul National University College of Medicine, REPUBLIC OF KOREA

## Abstract

Queen Nefertari, the favourite Royal Consort of Pharaoh Ramses II (Ancient Egypt, New Kingdom, 19th Dynasty c. 1250 BC) is famous for her beautifully decorated tomb in the Valley of the Queens. Her burial was plundered in ancient times yet still many objects were found broken in the debris when the tomb was excavated. Amongst the found objects was a pair of mummified legs. They came to the Egyptian Museum in Turin and are henceforth regarded as the remains of this famous Queen, although they were never scientifically investigated. The following multidisciplinary investigation is the first ever performed on those remains. The results (radiocarbon dating, anthropology, paleopathology, genetics, chemistry and Egyptology) all strongly speak in favour of an identification of the remains as Nefertari’s, although different explanations—albeit less likely—are considered and discussed. The legs probably belong to a lady, a fully adult individual, of about 40 years of age. The materials used for embalming are consistent with Ramesside mummification traditions and indeed all objects within the tomb robustly support the burial as of Queen Nefertari.

## Introduction

The tomb of Queen Nefertari (QV 66), the second Great Royal Wife of King Ramses II (lifetime ca. 1303–1213 BC), was discovered by Ernesto Schiaparelli (1856–1928) in the Valley of the Queens in 1904. Her burial had been looted in antiquity, so no trace of the original entrance had been preserved. Besides the famous wall paintings, a series of broken remains (e.g. a damaged pink granite sarcophagus, broken furniture, jars, shabtis, other various small items), a pair of sandals and two fragmented mummified legs (parts of tibiae and femora) are preserved. All these items and the human remains are currently housed in the (Museo Egizio Turin, Suppl. 5154 RCGE 14467) [1–3]. (Table A in S1 File).

**Fig 1:**
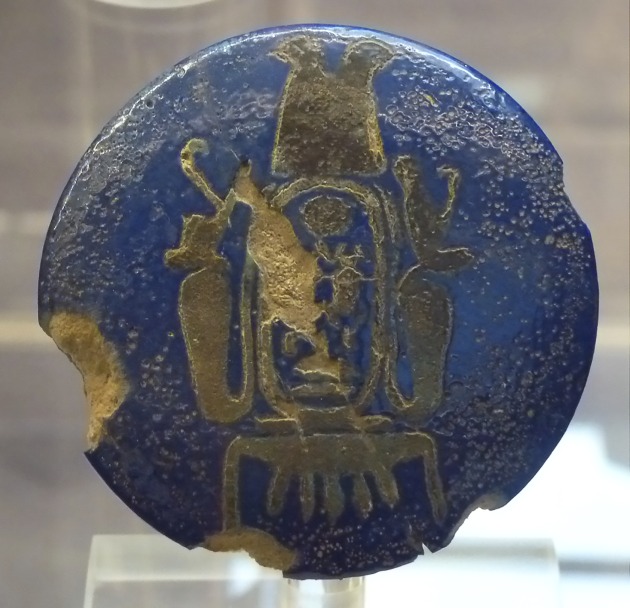
Knob head or pommel with the throne name (Kheper-Kheperu-Ra) of King Ay. Museo Egizio Turin Suppl. 5162.

**Fig 2:**
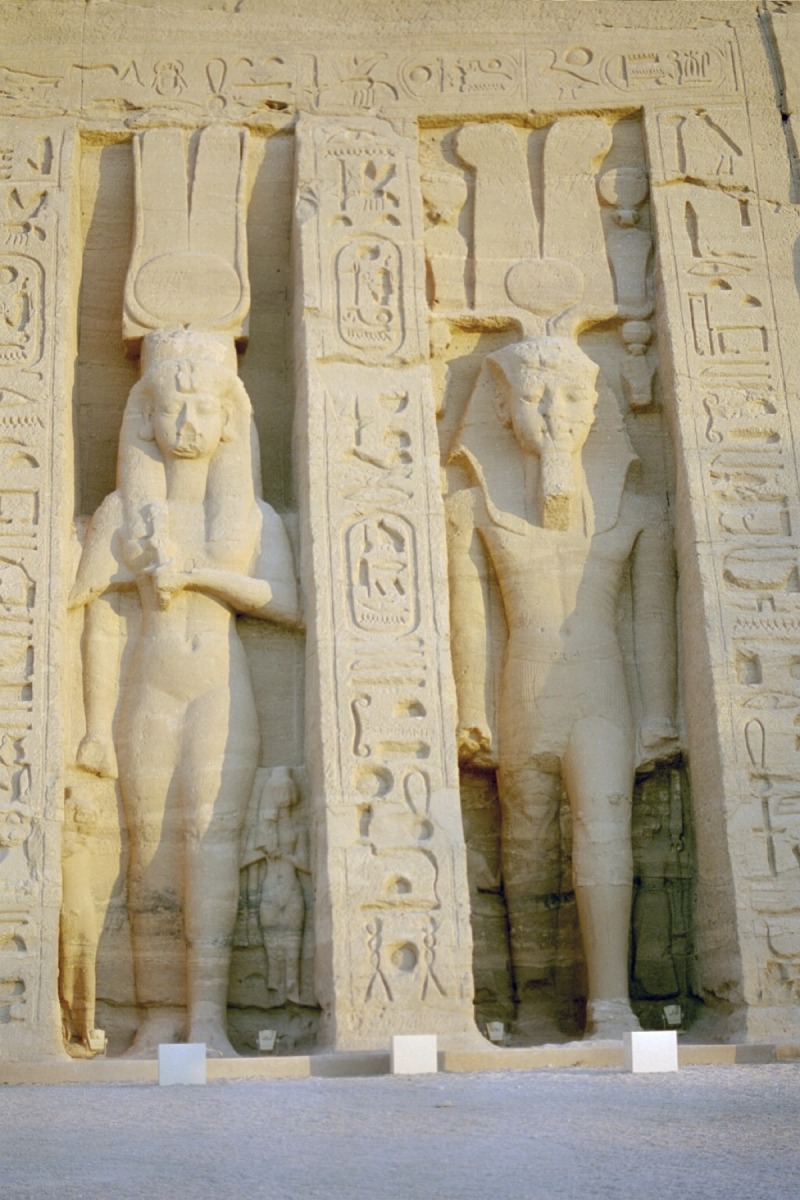
Abu Simbel, second rock temple dedicated to Nefertari, front: Nefertari’s statue shows the same size as Ramses II in order to demonstrate her status and importance.

Nefertari was the most beloved wife of King Ramses II and played an active role in foreign politics. Her ancestry is unknown. Based on the legible/decipherable inscriptions on a fragment of a faience knob head or pommel found in her tomb, speculations were raised [4,5]. The item carries the throne name ‘*Kheper-Kheperu-Ra*’ and, is, therefore, connected with King Ay [6], who ruled Egypt for a few years after Tutankhamun (Turin Mus. Egizio Inv. Suppl. 5162) [2,7]. However, Nefertari did not carry the title ‘*Daughter of a King’*, which suggests that she was probably not from the main royal line. Because of the chronology, it seems quite unlikely that she was King Ay’s daughter, perhaps she was Ay’s grand-daughter [6,8] (Fig 1). Other scholars emphasize that both Ramses II’s royal wives, Isisnofret [9] and Nefertari, had a non-royal background [10]. Nefertari married Ramses when he was crown prince during the reign of his father Sety I. The age at which Ramses II succeeded to the throne of Egypt is uncertain, possibly around his 25^th^ year [10]. Nefertari was then presumably the same age as her husband or slightly younger (ca. 20–25 years). She gave birth to four sons (Amun-hir-khepeshef, Pa-Ra-wenem-ef, Mery-Ra and Mery-Atum) and four daughters (Baketmut, Nefertari, Merytamun and Henuttaui). Within the succession line, Nefertari’s sons were always preferred to Queen Isisnofret’s although, in the end, the crown went to Merenptah, a son of Queen Isisnofret. Queen Nefertari, as attested by reliefs, attended the opening ceremony of the rock-cut temples of Abu Simbel in the year 24 of Ramses II’s reign (ca. 1255 BC) (Fig 2) [11]. After that event, she disappeared. She was absent at the Sed-festival of Ramses II’s 30^th^ regal year. She probably died around his 25^th^ year of reign [10]. As reconstructed from historical records, Nefertari probably reached an age of about 40 to 50 years (minimum 16 + 24 years or maximum 25 + 25 years) whereas Queen Isisnofret I died later, in year 34. Subsequently Ramses II married three of his daughters: Bint-Anat, Merytamun and Nebettaui [10].

### Aim

**Fig 3:**
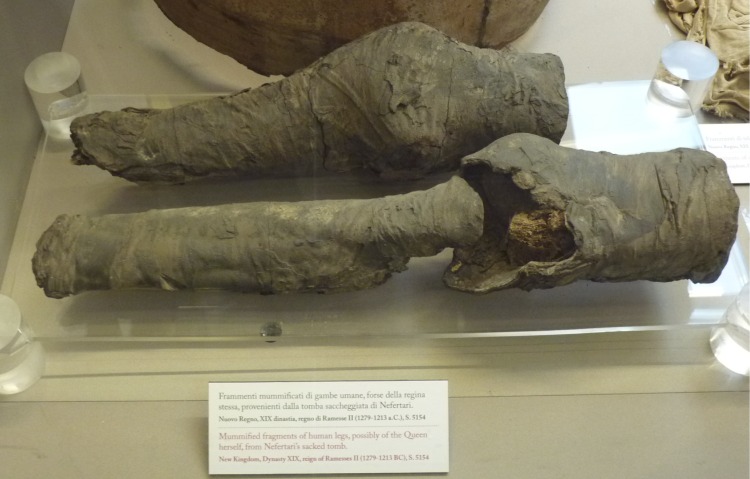
The mummified remains as shown in the 2014 exhibition in Museo Egizio Turin Suppl. 5154 RCGE 14467.

The aim of the research is to answer—via a multidisciplinary approach to a long historical debate–a complex question. Do the human remains found in the tomb belong to Queen Nefertari’s original burial? (Fig 3). The remains of Nefertari are considered as highly important for History and Egyptology since Nefertari is one of the most famous Queens of Ancient Egypt.

## Material

**Fig 4:**
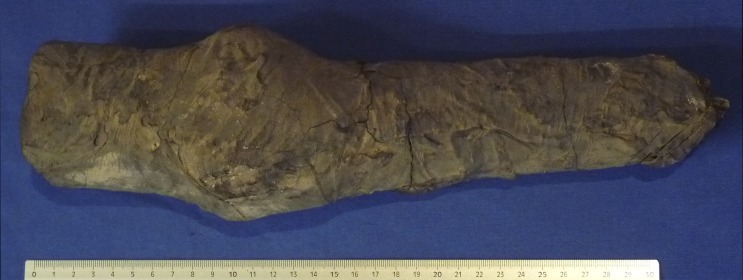
Long leg fragment (No. 1).

**Fig 5:**
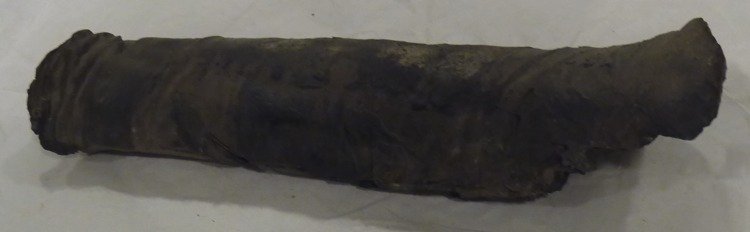
Medium sized part of tibia (Nr. 2).

**Fig 6:**
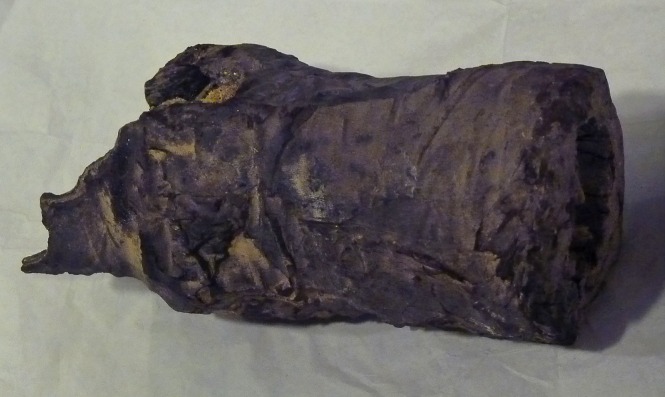
Short part of a femur (No. 3).

The human remains *Suppl*. *5154 RCGE 14467* consist of three separate mummified human body parts: A long leg fragment consisting of a distal femur part, patella and a proximal tibia part (Fig 4); a medium sized part of an incomplete proximal tibia (Fig 5) and a short part of a femur (Fig 6).

## Methods

### Ethics statement

Ethic clearance was not needed, since the studied remains are older than 3200 years and no living relatives are known.

The permission to investigate was given by the Egyptian Museum in Turin by the director Eleni Vassilika. Permission to perform the radiocarbon dating was given by the new director Christian Greco.

a) Number of specimen: Three parts, probably belonging to one individual. Finding location: Egypt, Valley of the Queens, tomb QV 66 of Queen Nefertari.

b) The remains are on permanent, public display in Turin, Mus. Egizio. S. 05154 RCGE 14467.

c) A detailed permission was not needed.

d) No permits by ethics committee were required for the described study. All investigations were done according to our code of ethics:

IEM Code of Ethics http://www.iem.uzh.ch/en/institute/iemcodeofethics.html

### Radiological assessment

The human remains were X-rayed *in situ* at Turin’s Museum, using a portable, state-of-the-art mobile digital X-ray (EXAMION PX 60 HF; max. output 3.2 kW, Voltage range 40–100 kV, Exposure range 0.4–100 mAs).

### Anthropometric reconstruction

To look for knee sizes, the condyle’s width was compared to modern samples (young females Cape Coloured) and to ancient samples from Metapontum (Italy 700–300 BC) [12,13] (Table A in S1 File). Different body height formulae (for modern and for prehistoric remains) [14–16] were used to investigate the individual’s stature. Comparison of the dimensions of QV 66 knees with those of modern poor Sub-Saharan African females was implemented [12].

### Comparison of knees with ancient and modern samples

Proportionality rule was applied to compare the ancient samples with the knees from QV 66. Testing of z-score (165 cm– 158 cm) / SD 7 = +1 SD (the less favourable data of 3^rd^ Intermediate Period) was implemented. By comparing the condyle’s width to Cape Coloured data, sex dimorphism characteristics were tested (studies from ancient Egyptian samples being wanted). The size of the QV 66 knees was then assessed for sex determination via a measurement of both condyles on scaled X-ray pictures (Table A in S1 File).

### Ancillary Egyptological analysis: the Sandals and Other Objects Found in Tomb QV 66

Sandals (also exhibited in the Turin museum) were found among other objects, such as the fragments of a stone sarcophagus of Nefertari bearing an inscription, 34 wooden shabtis bearing her name, two lids of coffers, fabric, broken pottery and fragments of wooden statues in Turin. Mus. Egizio (List B in S1 File). Almost all objects are either inscribed with the name of Nefertari or, at least, their styles link them to the 19^th^ Dynasty. The objects were philologically and epigraphically tested for consistency with the hypothesized time period and foot length can be used for forensic reconstruction of body height [17,18], using the regression equation for female [18] (87.906 + 3.165 x 24.5 = 165 cm).

Aside the obvious plundering of the tomb and its treasures there is no evidence for intended desecration of the Queen’s body.

Valid for chemical analysis, DNA analysis and radiocarbon dating: Small skin, muscle and textile biopsies (1cm x 1cm) were taken for biochemical analysis (GC-MS), ancient DNA as well as radiocarbon dating investigations.

### Chemical analysis

The amorphous organic residues impregnating the textile samples taken from the knee assemblage were chemically characterized and identified using gas chromatography (GC-MS). After initially grinding samples to a fine powder, a weighed amount of these ground samples (from 10–90 mg) was taken. These samples were then extracted with an appropriate volume (0.5–2 mL) of chloroform-methanol solution (2:1 v/v; 3x60 min sonication). After centrifugation (20 min, 1000 rpm) the supernatant solvent was removed from the residue and placed in a vial. The three extracts were combined and the solvent reduced by rotary evaporation. Following transfer of the combined extracts to a screw-capped vial, the remaining solvent was removed by evaporation under a gentle stream of nitrogen at 40°C. The residue was reweighed to give total lipid extracts (TLE). The TLEs were trimethylsilylated using N,O-bis(trimethylsilyl)trifluoroacetamide (Sigma-Aldrich Chemical Co., St Louis, MO, USA) containing 1% of trimethylchlorosilane (50 μl, 70°C, 1 hour). Excess BSTFA was then removed under a gentle stream of nitrogen and the derivatized sample redissolved in dichloromethane and analyzed by GC-MS. GC-MS analysis of the total lipid extract of each sample was performed on a a Hewlett-Packard 5890 Series II gas chromatograph fitted with a split injector (325°C) interfaced to a Trio 1000 mass spectrometer (electron voltage 70eV, filament current 200 uA, source temperature 170°C, interface temperature 325°C). The acquisition was controlled by Windows based MasSpecII32 Data System, in full scan mode (35–650 amu). Separation was performed on a fused silica capillary column (30 m x 0.25 mm i.d) coated with 0.25 um 5% phenyl methyl polysiloxane (DB-5). Initially the GC was held at 40°C for 5 minutes and then temperature programmed from 40°C-350°C at 8°C min and held at final temperature for 20 minutes (total time 63.75 minutes), with helium as the carrier gas (constant flow 1 ml/min, initial pressure of 45 kPa, splitless injection 1 min). Identification of compounds was achieved on the basis of both their mass spectra (NIST Mass Spectral Database and reference compounds) and relative retention times (relative retention indices (RRIs)).

### DNA analysis

The analyses were performed at the dedicated ancient DNA laboratory at the Institute of Evolutionary Medicine of the University of Zurich. For genetic analysis the samples were cleaned using a 1% bleach solution to remove contaminating DNA from modern individuals that had handled the mummified remains and samples before air drying and crushed in a SPEX freezer mill (6770) to form a fine powder. The DNA was released from the bone powder by decalcification for 48 hours (12 hours 55°C and 36 hours at room temperature) in a 0.45M EDTA (Ethylenediaminetetraacetic acid) solution with 100mg Proteinase K added to remove excess proteins and inhibit enzymatic activity. The DNA was released from the soft tissue using the extraction buffer (10mM Tris-HCl, 10mM NaCl, 5mM CaCl2, 2,5mM EDTA, 2% SDS, 40mM DTT and 100mg/ml Proteinase K) for 18 hours at 55°C. The supernatant, containing released DNA, was then subjected to a Phenol-Chloroform extraction to remove any further proteins (mixed twice with 25:24:1, phenol:chloroform:isoamyl alcohol, and the DNA containing supernatants removed, final wash with chloroform), before being concentrated with a modified QiaQuick PCR purification method (final elution incubation at 37°C for 5 minutes to maximise DNA yield). DNA extracts were subjected to conventional PCR amplification of the HVRI of the mtDNA D-loop with four overlapping primer sets and to a sexing assay using real-time PCR as previously described [19] Each extract was analyzed for mtDNA data twice and for sexing data three times, and three non-template extraction controls and reagent blanks were processed in parallel with each PCR.

### Radiocarbon dating

Original sample of mummified tissue taken from the interior compartment of femura and tibiae contained 108 mg of material. A sample of 79.1 mg was taken for analysis and treated in a soxhlet system. A sequence of solvents (chloroform, hexane, acetone and ethanol) was used to remove resins and waxes [20]. The remaining sample with a mass of 61.7 mg underwent modified acid base treatment [21]. The short treatment in room temperature (instead of 60C) was applied because the material underwent rapid dissolution. Only 20% (i.e. 13.5 mg) of the sample remained after ABA. The remaining material was weight into tin cups for a combustion Elemental Analyser and subsequent graphitization [22] two targets were prepared from the material: one contained mainly powder of the tissue and the second the remaining of the sample. These were then analysed using MICADAS, which is a dedicated ^14^C AMS instrument at the AMS facility, ETH Zurich [23]. The measured ^14^C content (F14C) was normalised to the standard Oxalic Acid 2 corrected for blank values and isotopic fractionation using delta ^13^C measured on graphite see Hadjas 2008 [21]. Radiocarbon ages were calculated following the convention of Stuiver and Polach 1977 [24]. OxCal program [25] and INTCAL13 [26] data set were used to calibrate to calendar ages.

## Results

### Radiological assessment

**Fig 7:**
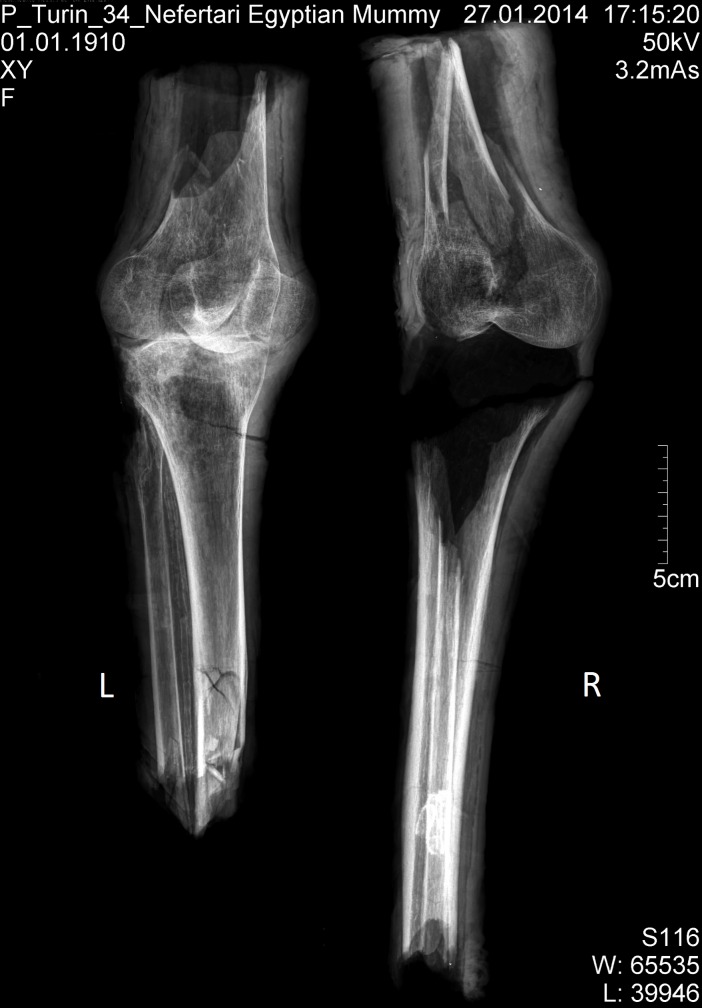
X-ray; arranged as seen in Fig 3.

**Fig 8:**
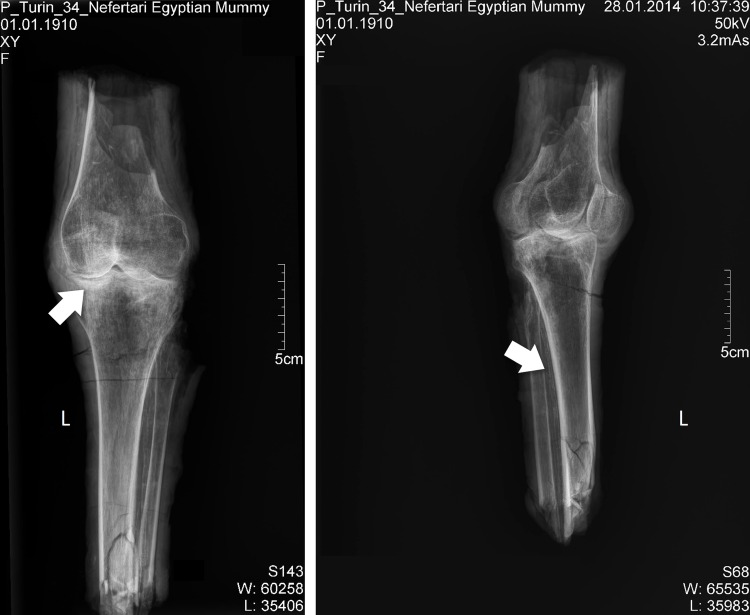
Left: X-ray left knee; the arrow marks the minimal signs of arthritis. Right: lateral view; the arrow points to the calcification of the arteriae tibiales.

**Fig 9:**
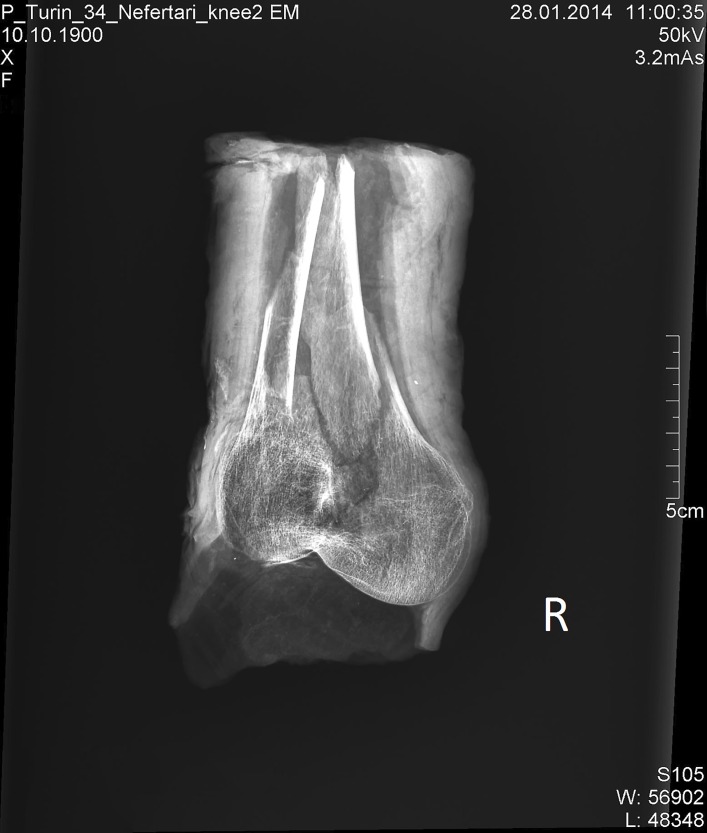
X-ray of right distal femur, pa.

**Fig 10:**
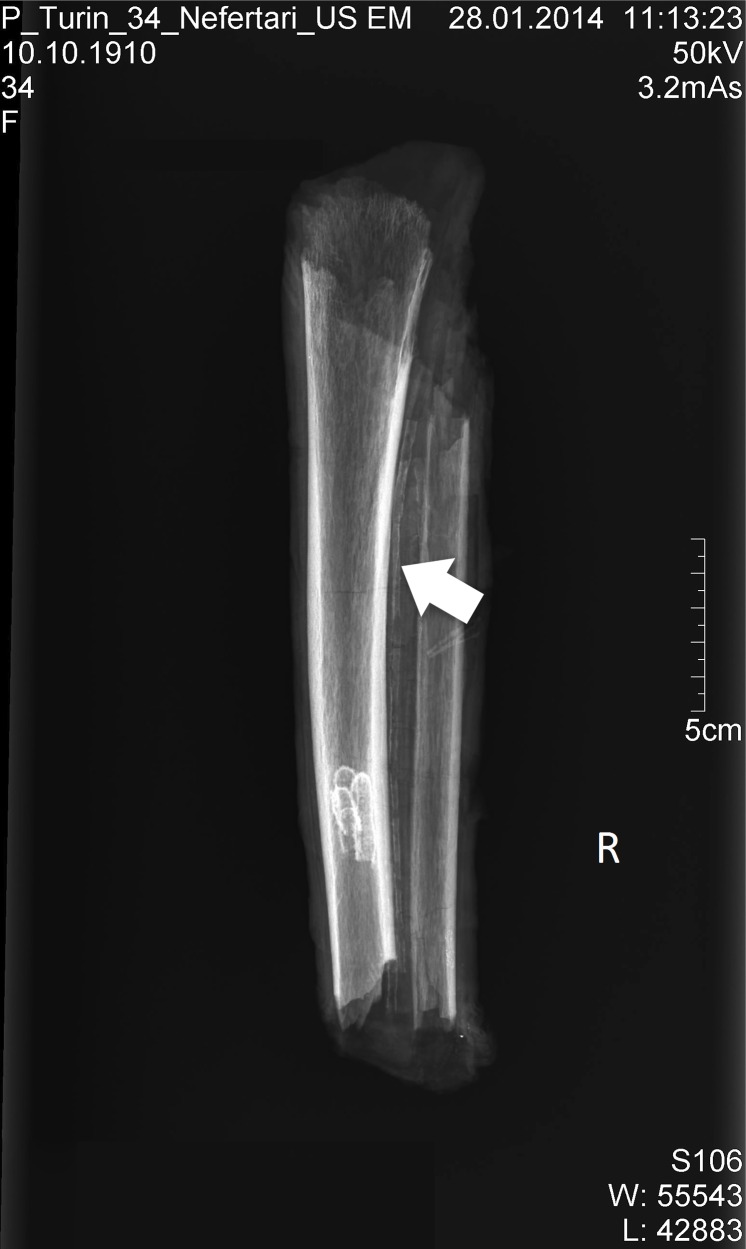
Fragments of left tibia and fibula; the arrow points to the calcification of the arteriae tibiales.

The X-rays confirmed the presence of a pair of human knees, with distal part of femur, proximal part of tibia and fibular bone as well as the patella (Figs 7–10). It is sensible and a somewhat likely hypothesis that the remains actually belong to a single individual as also suggested by close visual inspection which, based on colour and texture, shows how the remains appear to be those of a single individual (Figs 3–6). This however cannot be proved with absolute certainty. The remains show massive, probably post mortem, multiple impacted fractures. While the femur does not show any fractures, the tibia on the contrary shows a fracture proximally and is multiply fractured at the distal end. The knee joint shows a narrowed joint space, a finding that is commonly observed in mummies and that does not necessarily imply underlying pathologies. The bandage layers can be differentiated on the X-rays. X-ray of the leg split in two separate parts show a right distal femur with multiple fractures (Fig 7) with surrounding soft tissue as well as bandage layers. This part is only fragmentarily preserved; only the metaphyseal parts of the left tibia and fibula are preserved (Fig 8). Surrounding soft tissue and bandage layers are visible. No prominent bone pathology (e.g. a tumorous lesion) could be spotted. The bone architecture is indeed generally normal (Figs 7–10). All epiphyses are fused, which implies that the remains belong to an adult individual. The X-rays revealed instead a very thin cortical thickness (ca. 1–2 mm) for which several differential interpretations may thus be given: minimal osteoathrosis, osteoporosis, vitamin D deficiency-caused osteomalacia (e.g. if the individual was secluded from sunlight), or disuse osteopaenia from cerebral palsy or other causes. If, in the absence of further clinical evidence, most of these pathologies are excluded and only minimal osteoarthrosis or osteopaenia are considered, then the eventuality that the individual underwent minimal physical labour as a consequence of a high status, characterised by predominantly indoor life can be proposed. No definitive solution however exists to this problem. In addition, in the left knee, it is possible to see what may be a calcification in the arteriae tibiales (anterior and posterior). This finding can be caused by arteriosclerosis or media calcinosis (Mönckeberg’s sclerosis). Both differential diagnosis suggest an elderly person. Thus, we assume as one of the possibilities that the knees belonged to an individual older than 40 years. The accumulated evidence could point to an individual between 40 and 60 years old.

### Anthropometric reconstruction

Both knee condyles show a ca. 83–85 mm width if mummified soft tissues are included and ca. 79–80 mm if only the bone is considered. A condyle width of ca 83–84 mm indicates that QV 66 knees were slightly slimmer than those of the younger and poorest women from Sub-Saharan Africa. There is no formula to re-calculate knee width from living to dead, only an estimate the greatest difference would be ca. 1.5 mm in knee width between living and dead persons [12,27,28]. Moreover, it was also possible to determine—acknowledging a certain degree of uncertainty—that the bones found in QV66 belonged to an individual whose stature ranged between 165 cm and 168 cm (Table A in S1 File).

### Comparing knees with ancient and modern samples

Assessment of the size of the QV 66 knees revealed them to be female with a 90% likelihood. With a single exception, the knees from QV 66 belong to an individual taller than e.g. the average ancient Greek women’s range (Table A in S1 File). From the size and proportion of the knees, the most likely body height of QV 66 female was determined to be 165 cm (+/- 2.5 cm). Compared with data about women from the New Kingdom (average 156 cm) and 3^rd^ Intermediate Period (average 158 cm), she was taller than the average Egyptian woman [29]. The QV 66 female was approximately one Standard Deviation taller than average (or taller than 84% of the women of her time). The estimated height of ca. 165 cm is confirmed independently by the calculation of foot size and body height reconstruction obtained from the sandals found in the tomb, which, indeed, belonged to an individual of ca. 165 cm (see below). Compared to e.g. ancient Greek females QV 66 female is 95% above the ancient Greek female range and close to the average male (she is equal in height to ancient Greek and Egyptian men) [13,29].

### Ancillary Egyptological analysis: the Sandals and Other Objects Found in Tomb QV 66

Only the faience knob with the name of King Ay found in tomb QV 66 belongs to the late 18^th^ Dynasty and predates Ramses II and Nefertari by perhaps two generations. The poor quality of the shabtis was also a matter of speculation as they seemed ill-fitting for a burial of a great Queen [8]. A fragment of a golden object with the name of Nefertari was discovered in 1988 when the tomb was restored [8]. Other fragments of jewellery without a provenance but bearing the name of the queen are also known. They may also come from QV 66 (List B in S1 File) [8].

**Fig 11:**
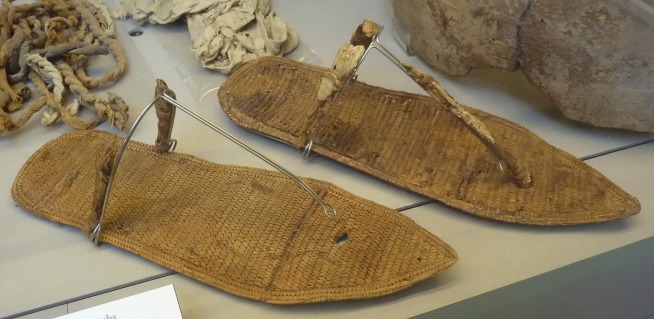
Sandals from tomb QV 66. Museo Egizio Turin Suppl. 5160 RCGE 14471.

The sandals are made of sewn fibre and they belong to the group of type C sandals (Veldmeijer´s classification): type C variation 1; the front strap is Type 3 and back strap is type 2 [30,31]. The style is typical of the 18^th^– 19^th^ Dynasties [32,33]. The sandals from QV 66 show some wear caused by the movement of the foot on the dorsal (upper) side, the ventral side could not be studied due to mounting on a display panel (neither by Veldmeijer, nor by the authors of the present study). The sandals measure 29 cm in length and 10 cm in width (Fig 11). Type C has a pointed, slightly upturned toe pointing to a modern shoe size of 39–40, if one only counts the length used by the foot, indicated by the imprints and the subtraction of the pointed end [34]. Furthermore the model clearly indicates the position of the big toe, with visible marks of the size, especially on the left sandal: It can be deduced, with a certain reservation, that the sandals’ owner had a body height ranging c. 165 cm using forensic methods [17]. Veldmeijer described the sandals as those of Queen Nefertari [30]. The fine quality manufacture and high quality of the sandals speaks in favour of royal footwear. Thus it is widely accepted, that all objects found in QV 66 seem to be part of the original burial of Queen Nefertari, broken by ancient tomb robbers [7].

### Chemical analysis (data on embalming agents)

**Fig 12:**
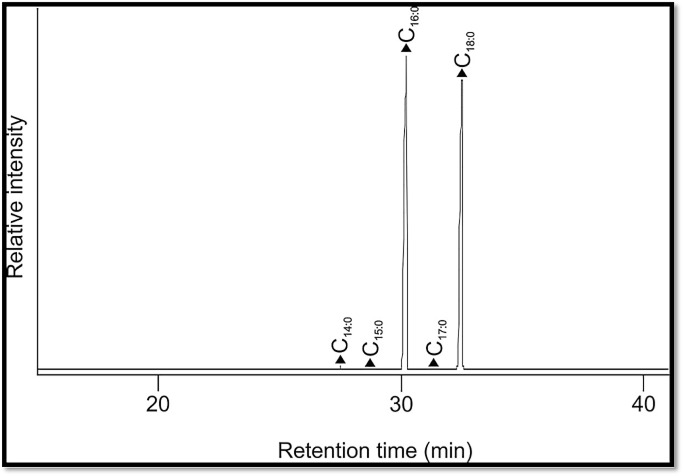
Reconstructed gas chromatography-mass spectrometry (GC-MS) total ion chromatogram (TIC) of the trimethylsilylated total lipid extract of ‘resin’/linen wrapping from left long leg fragment. Peak identities (‘n’ indicates carbon chain length; where shown, i indicates degree of unsaturation): filled triangles, C_n:i_ indicates fatty acids.

The results of the chemistry of the embalming agents suggest a date earlier than the 3^rd^ Intermediate Period, which is consistent with the evidence for the mummification materials and methods detailed below: the absence of bitumen is consistent with a New Kingdom date since it does not appear in balms from mummies until 900 BC [35,36]. The use of bitumen, and more liberal employment of tree resins in the embalming recipes, is seen in 3^rd^ Intermediate Period mummies and later, the use and proportion of both in relation to the plant oil/animal fat base increasing over time with greatest use during the Ptolemaic and Roman Periods [35,37–39]. Bitumen was not detected in any of the samples from the knee assemblage despite selectively monitoring for the presence of hopanes (m/z 191) and steranes (m/z 217) characteristic of a true natural bitumen [40]; Constituents of coniferous and non-coniferous resins were also not detected. The biomarkers for both these natural products are highly resilient and so can be expected to survive in a burial environment such as QV66 if they were originally present (Fig 12). This is consistent with these samples being largely from the outer layers of wrappings where oils or fats are usually the main or only ‘embalming agent’ during the New Kingdom, and are used to convey religious, political and cultural identities at this time [37]. In this context, it is notable that the samples from the mummified knees all revealed a non-human animal fat as the source of the embalming agents applied liberally to their linen wrappings, with all parts of the knee ‘assemblage’ showing a very similar lipid (fat) profile suggesting a likely common origin, i.e. the same individual. The same non-human animal fat, most likely a ruminant fat, constituting the embalming agent in the outer wrappings from all three parts of the knee assemblage, combined with the absence of evidence for a natron bath being employed and other aspects of the mummification, suggest a 19^th^ or 20^th^ Dynasty date for the mummification. Massive sub-cutaneous stuffing, the characteristic of the 3^rd^ Intermediate Period, is not visible.

### DNA analysis

Mitochondrial sequences were only obtained from Primer Set 3, and only from the left fibula (bone) and the upper right (bone and soft tissue) samples. All samples showed multiple mtDNA sequences, with the soft and bone tissue from the upper right sampling area showing different sequences from each other. This indicated that there are at least two contamination events in these samples. The sexing assay showed a weak amplification of the X-chromosomal target in the left fibula bone sample (twice) and the left femur soft tissue sample (once), and one strong signal in the upper right soft tissue sample. The clear evidence of allelic drop-out, together with the evidence of contamination from the mtDNA data, means that no conclusions can be drawn on these data. The inappropriate genetic behaviour exhibited in these samples (for example, strong amplification of nuclear DNA with no mtDNA amplification as seen in the upper right soft tissue sample) is further evidence that these samples are not suitable for further DNA analysis.

### Radiocarbon dating

**Fig 13:**
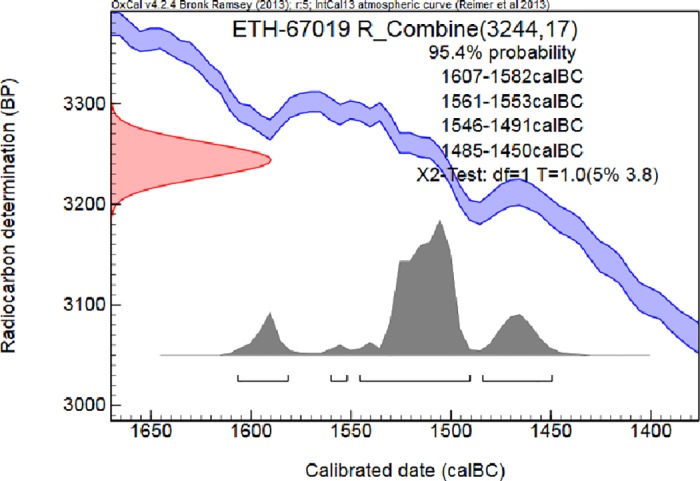
Calibrated result of the radiocarbon dating.

The radiocarbon ages obtained on the 2 targets are in a very close agreement (ETH-67019.1: 3261±24 BP; ETH-67019.2: 3227±24 BP). The combined age of the sample is 3244±17 BP, X2-Test: df = 1 T = 1.0(5% 3.8). The calibration of this combined radiocarbon age results in a wide range of calendar ages. In some cases due to the shape of the calibration curve in the region of interest, the age of the sample falls into a period, where more precise information about the true age cannot be given [20]. Such is the case of this sample (Fig 13) and all the intervals between 1607BC and 1450 BC (95.4% conf. level) has to be considered.

OxCal v4.2.4 [25]; r:5. IntCal13 atmospheric curve [26]

ETH-67019 R_Combine (3244,17)

68.2% probability    1531BC (65.1%) 1497BC

                    1469BC (3.1%) 1465BC

95.4% probability    1607BC (9.3%) 1582BC

                    1561BC (1.2%) 1553BC

                    1546BC (69.0%) 1491BC

                    1485BC (15.8%) 1450BC

All results fall in the time of the late 2^nd^ Intermediate Period and the New Kingdom (Fig 13).

## Discussion of the Different Hypotheses

The objects and human remains found in tomb QV 66 provide us with information which allows a contextualization of the findings and to access to their likelihood.

### Discussion on the radiocarbon dating of the remains

The obtained radiocarbon age is older than historic date of the tomb QV 66 but a discrepancy between ^14^C based chronology and Egyptian calendar has been debated ever since Minoan Eruption of Thera was dated by ^14^C (ranging 2σ 1663–1599 B.C.) [41,42]. In addition, fish diet could have possible effect on the ^14^C age of the tissue as discussed in the study of mummified Ibis [43]. Such discrepancies between ^14^C dates and assumed chronological models are observable for several time periods [44]. The results appear slightly older than the assumed lifespan of Queen Nefertari (early 19^th^ Dynasty). The potential contamination sources could be older embalming agents used for mummification as well as intruding sediment during the recorded several mudslides in antiquity [8]. Such potential contamination would make the sample appear older. Although the old (stored) conservation agents cannot be excluded the treatment of samples removed potential contamination with carbonates and humic acids, which could originate from sediments. Furthermore, the geography of the valley and the location of tomb QV 66 make it unrealistic that older remains were washed in uphill. Rather a later dating of the remains in QV 66 would be problematic for identification as Nefertari.

### Hypothesis 1: The mummified legs belong to Queen Nefertari

Reconstruction is based on ancient Egyptian funerary customs and recorded evidence found in QV 66. Nefertari died aged 40 to 50 years after the 24th year of Ramses II’s reign and was embalmed. Her mummy was decorated with funerary jewellery bearing her name as the deified Osiris (Boston, Museum of Fine Arts Inv. 04.1954, Inv. 04.1955, Inv. 04.1956). Her mummy was placed in gilded wooden coffins (splinters were found in QV 66). The coffins were placed in a stone sarcophagus (Turin, Mus. Egizio S. 5153 RCGE 17494) bearing her name. The niches in the burial chamber were equipped with magical bricks (Turin Mus. Egizio S. 5163 RCGE 14473). Statues of Gods made of black-coated wood were placed in her tomb (Turin Mus. Egizio S. 5202 RCGE 14477) along with other funerary goods, some of which bear her name (coffers: Turin Mus. Egizio S. 5198, RCGE 14474, S. 5199 RCGE 14475). The reason why the faience knob or pommel inscribed with Kheper-Kheperu-Ra’s (= King Ay) name was found in QV 66 remains a mystery. It is possible that Nefertari was a surviving descendant of the 18^th^ Dynasty royal family. The tomb robbers smashed the stone sarcophagus, pulled the coffins out and ripped the mummy into pieces. The remains were thrown on the ground; the funerary equipment was plundered and only the wooden, clay and stone objects were left behind. Some of the funerary jewellery was lost during the looting. Later water intrusions badly damaged the tomb leaving a layer of debris over the objects. The basic anatomical observations and the mummification methods and materials are consistent with a high status burial from the 19^th^ Dynasty.

### Hypothesis 2: The remains Nefertari and one of her daughters

Following a second hypothesis, the tomb looters took away all objects belonging to Nefertari´s children and only those of Nefertari remained in the debris. The tomb robbers smashed the stone sarcophagus, pulled the coffins out and ripped the mummies of Nefertari and her children into pieces. As a matter of fact some of Nefertari´s daughters were buried in their own tombs in the Valley of the Queens: Merytamun in QV68 (her broken sarcophagus is preserved in Berlin) and Nebettaui in QV60. Even if one of her daughters, Baketmut or/and Henuttaui may have been buried at the side of their mother, there is no archaeological indication of an additional burial in QV 66. The likelihood of this hypothesis is considerably low.

### Hypothesis 3: A secondary burial

The radiocarbon dating, chemistry and archaeology rule out a later burial in the 3^rd^ Intermediate or Late Period entirely.

### Hypothesis 4: The remains are washed in from an earlier burial

The results from the radiocarbon dating offer the possibility that remains from a burial of the 17^th^ or 18^th^ Dynasty were washed in the tomb after it was open. Archaeological material found does not support such a hypothesis (e.g. material from other time periods and inscriptions naming other individuals). Tomb QV 66 is on higher ground at the side of the Valley of the Queens, while the burials from the 17^th^ and 18^th^ Dynasty are on lower ground, mostly at the bottom of the valley. Mudslide and heavy rains would have washed remains out of the valley but unlikely upwards and towards the end of the valley.

## Conclusion

The first hypothesis seems to be the most credible and realistic and is coherent with the findings of the excavators and with the inscriptions found on the funerary objects. Thus, the most likely scenario is that the mummified knees truly belong to Queen Nefertari. Although this identification is highly likely, no absolute certainty exists. A list of default criteria was made to test the likelihood of the first hypothesis (Table 1). Certain default criteria were not found, which would exclude the identification of the knees as those of Queen Nefertari (default criteria by chemistry or aDNA with reservation). The fitting criteria are in the majority (Table 1).

**Table 1:** Likelihood (default criteria to exclude Nefertari).

Question	Result	Requirements for Nefertari	Fitting Inconclusive or Default
Genetic sex test	undefined, no Y-chromosome found	Female	Inconclusive
Anthropometric reconstruction	Tall female, very slim	female, perhaps tall, as she is usually represented in the Ramesside art	Fitting
Genetic profile		No genetic data from Ramses II or her children available (may remain inconclusive for the time being)	Inconclusive
Radiocarbon dating	New Kingdom, c. 3447 BP	Minimum 3200 BPA dating later than 19^th^Dynasty would point to a secondary burial (default)	Older than expected, not a default
Age assessment	Adult 40–50 years	40 to 50 years	Fitting
Chemistry of the embalming agents	Chemistry of all parts is the same. They belong most likelyto one individual. Thechemistry suggests New Kingdom and 19-20^th^ Dynasty	Should be New Kingdom and royal. No use of Bitumen.	Fitting
Mummification style	No natron bath, no stuffing,Dry natron, royal quality	No natron bath, no stuffing visible, good quality	Fitting
Archaeology	Tomb broken in antiquity	No indication of a burial post-dating the Ramesside Dynasty	Fitting
Sarcophagus	Name of Nefertari inscribed	19^th^ Dynasty Sarcophagus	Fitting
Magic bricks	Made for Nefertari, her name is inscribed	Made for Nefertari	Fitting
Shabtis	Made for Nefertari, her name is inscribed	Made for Nefertari	Fitting
Jewellery	Funerary jewellery with her name inscribed	Jewellery of royal quality, with her name inscribed. Funerary jewellery	Fitting
Furniture	Made for Nefertari, her name is inscribed	Made for Nefertari	Fitting
Wooden black statues	Wepwawet or Anubis	Typical funerary statues in royal burials	Fitting
Sandal	Size 39, Type Veldmeijer C Var. 1 (later 18^th^ to 19^th^ Dynasty) [30]	Footwear in style of 19^th^ Dyn.	Fitting
Pommel (of Sceptre or furniture)	Name of King Ay	No object is later than the time of Ramses II	Fitting

From 16 criteria are 14 classified as fitting and 2 as inconclusive. A certain default was not found.

## Supporting Information

S1 File Habicht et al. Nefertari Supplementary Material Online PLOSone.pdf. **Table A in S1 File: Ancient Greek anthropometric data** (Metapontum, Henneberg and Henneberg, 1998) with subsequent proportional calculations of body height as for QV 66.**List B in S1 File: Objects from tomb QV 66** now in Turin, Mus. Egizio and Boston, Mus. of Fine Arts.
(DOCX)
