# Supplementary material for: Queen Nefertari, the Royal Spouse of Pharaoh Ramses II: A Multidisciplinary Investigation of the Mummified Remains Found in Her Tomb (QV66)
Source: PLoS One. 2016 Nov 30;11(11):e0166571. doi: 10.1371/journal.pone.0166571 (PMC5130223; doi:10.1371/journal.pone.0166571)
Supplement: S1 File — Table A in S1 File: Ancient Greek anthropometric data (Metapontum, Henneberg and Henneberg, 1998) with subsequent proportional calculations of body height as for QV 66.List B in S1 File: Objects from tomb QV 66 now in Turin, Mus. Egizio and Boston, Mus. of Fine Arts. (DOCX) [file pone.0166571.s001.docx]

**Queen Nefertari, the Royal Spouse of Pharaoh Ramses II: A Multidisciplinary Investigation of the Mummified Remains Found in Her Tomb (QV66)**

Michael E. Habicht ^1^, Raffaella Bianucci ^2, 3,^ , Stephen A. Buckley ^4,5^ , Joann Fletcher ^4,5^, Abigail S. Bouwman ^1^, Lena M. Öhrström ^1, 6^, Roger Seiler ^1^, Francesco M. Galassi ^1^, Irka Hajdas ^7^, Eleni Vassilika ^8^, Thomas Böni ^1^, Maciej Henneberg ^9^, Frank. J. Rühli ^1 *^

^1^ Institute of Evolutionary Medicine, University of Zurich, Zurich, Switzerland.

^2^ University of Turin, Department of Public Health and Paediatric Sciences, Legal Medicine Section, Turin, Italy.

^3^ UMR 7258, Laboratoire d’Anthropologie bio-culturelle, Droit, Etique & Santé (Adés), Faculté de Médecine de Marseille, Marseille, France.

^4^ University of York, Department of Archaeology, York, UK

^5^ BioArCh, Departments of Archaeology, Biology & Chemistry, University of York, UK.

^6^ University Hospital Zurich, Department of Radiology, Zurich, Switzerland.

^7^ Ion Beam Physics. Labor f. Ionenstrahlphysik (LIP), ETH Zürich, Zurich, Switzerland

^8^ Fondazione Museo Egizio of Turin, Turin, Italy.

^9^ Medical School, University of Adelaide, Adelaide, Australia.

* [frank.ruehli@iem.uzh.ch](mailto:frank.ruehli@iem.uzh.ch)

**Supplementary Material**

**A:** Ancient Greek anthropometric data (Metapontum, Henneberg and Henneberg, 1998) with subsequent proportional calculations of body height as for QV 66.

| **Sample** | **Condyle width (one, both sides)** | **Femur length** | **Tibia length** | **height (Trotter Gleser female white)** | **Corrected for QV 66** | **Height (Trotter Gleser female black African)** | **Corrected for QV 66** | **Height (Pearson 1899 female)** | **Corrected for QV 66** |
| --- | --- | --- | --- | --- | --- | --- | --- | --- | --- |
| Female | 77 mm | 410 mm | 326 mm | 155.5 cm | 156.5 cm | 155.74 cm (femur) 156.1 cm (tibia) | 161.8 cm | 152.0 cm | 157.9 cm |
| Female tomb 99 | 68 and 69 mm | 409 mm | 354 mm | 155.4 cm | 181.5 cm | 155.5 cm (femur) 163.0 cm (tibia) | 181.6 cm | 155.1 cm | 181.0 cm |
| Female tomb 100 | 80 mm | 452 mm | No data | 165.7 cm | 165.7 cm (no correction 1:1) | 165.3 (femur) | 165.3 cm | 160.8 cm | 160.8 cm |
| Female | 70 mm | No data | 343 mm | 161 cm | 162.1 cm | 160.3 cm (tibia) | 171.8 cm | 155.4 cm | 166.6 cm |
| Female | 72 and 74 at femur head 70 and 70 at tibia head | 393 and 394 mm | 330 mm both | 153.8 cm | 168.5 cm | 151.9 cm (femur) 157.1 (tibia) | 166.4 cm 168.3 cm | 150.6 cm | 165.1 cm |
| Female | 75 mm femur heads 71 tibia heads | 427 mm | 354 and 352 mm | 161.6 cm | Femur 172.4 cm Tibia 170.7 cm | 159.6 cm (femur) 162.8 cm (tibia) | 170.3 cm171.9 cm | 157.0 cm | 167.4 cm |

**B:** Objects from tomb QV 66 now in Turin, Mus. Egizio and Boston, Mus. of Fine Arts:

1. Sandals, fibre, 29 x 10 cm, Mus. Egizio S. 5160 RCGE 14471. Sewn sandals, Veldmeijer Type C, var. 1 [1].
2. Djed-Amulet, 13 cm, Mus. Egizio S. 5163 RCGE 14473. It is a magical brick, placed in a niche as magic protection of the burial. This burial object is a strong argument for a burial of Queen Nefertari in QV 66.
3. Fragment of Djed-Amulet, 15 cm, Mus. Egizio S. 5204 RCGE 14479.
4. Fragments of a stone sarcophagus with inscription, 40 x 110 x 265 cm, pink granite, Mus. Egizio S. 5153 RCGE 17494.
5. Lid of a black coffer, 26 x 19 cm, with inscription and name of Nefertari, sycamore wood, Mus. Egizio S. 5198, RCGE 14474.
6. Lid of a coloured coffer, 13.5 x 9.5 cm, with inscription and name of Nefertari, sycamore wood, Mus. Egizio S. 5199 RCGE 14475.
7. Fragments of a rope, fibre, Mus. Egizio S. 5157 RCGE 14469.
8. Fragments of fabric, fibre, Mus. Egizio S. 5256 RCGE 14468.
9. Fragment of a vase, calcite, Mus. Egizio S. 5211 RCGE 14481.
10. Mummified remains, three parts of knees, Mus. Egizio S. 5154 RCGE 14467.
11. Pommel of a sceptre or a coffer knob, faience. Cartouche Kheper-Kheperu-Ra (King Ay), Mus. Egizio S. 5162 RCGE 14472. Late 18^th^ Dynasty.
12. 34 shabtis, sycamore wood and bitumen, with inscription and name of Nefertari, Mus. Egizio S. 5164- 5197 all: RCGE 13509.
13. Fragment of a wooden statue, 32 cm, Mus. Egizio S. 5202 RCGE 14477. Probably the tail of an Anubis- or Wepwawet-statue, similar to the statue found in Tutankhamun´s tomb (Cairo JE 61444, Carter No. 261)
14. Fragment of a wooden statue of an Ibis, 25 cm, Mus. Egizio S. 5201 RCGE 14477. Similar statues of Thoth as an ibis are known from Horemheb and other Kings.
15. 4 fragments of blue glazed vases, Mus. Egizio S. 5210, RCGE 14481. The style of ceramic points to the 19^th^ Dynasty.
16. Jar, clay, Mus. Egizio S. 5206 RCGE 13507. The style points to the New Kingdom.

Other shabtis from QV 66 are kept in Toronto and Cairo and Boston. Albert M. Lithgoe acquired for the Museum of Fine Arts three fragments of jewellery and shabtis, allegedly from her tomb in 1904 [2].

- Shabti, Boston, Museum of Fine Arts Inv. 04.1766
- Shabti, Boston, Museum of Fine Arts Inv. 04.1767
- Shabti, Boston, Museum of Fine Arts Inv. 04.1768
- Shabti, Boston, Museum of Fine Arts Inv. 04.1769
- Part of a gold bracelet of Nefertari, Boston, Museum of Fine Arts Inv. 04.1954. The inscription “Great royal wife, Nefertari-beloved-of-Mut, justified” points to a jewel specially made for the funeral.
- Part of a gold bracelet of Nefertari, Boston, Museum of Fine Arts Inv. 04.1955. The inscription describes Nefertari as “Osiris”, thus it was also specially made for the funeral.
- Gilded Lotus ear-ring, Boston, Museum of Fine Arts Inv. 04.1956. Similar earrings are seen on the wall paintings in tomb QV 66 depicting Queen Nefertari.

Another object might also came from QV 66:

- Ivory Unguent Box with cartouches of Ramses II and Nefertari, 3 cm high, 5 cm diameter. New York, Metropolitan Museum, Inv. 26.7.1291. Acquired in 1926 [2]

**Supplement references**

1. Veldmeijer AJ. Studies of Ancient Egyptian Footwear. Technological Aspects. Part VI. Sewn Sandals. In: Ikram S, Dodson A, editors. Beyond the Horizon Studies in Egyptian Art, Archaeology and History in Honour of Barry J Kemp. Cairo: The Supreme Council of Antiquities; 2009. pp. 554–580.

2. Willeitner J, Schmidt HC. Nefertari. Gemahlin Ramses II. Mainz: Philipp Von Zabern; 1997.
